# Bilateral Moraxella Keratitis in a Healthy Person after an Episode of Conjunctivitis: A Case Report

**DOI:** 10.31729/jnma.6170

**Published:** 2021-05-31

**Authors:** Leena Bajracharya, Rachana Singh Rana

**Affiliations:** 1 Tilganga Institute of Ophthalmology, Gaushala, Kathmandu, Nepal

**Keywords:** *conjunctivitis*, *moraxella keratitis*, *pseudomembranous*

## Abstract

Moraxella species are gram-negative diplobacilli and are rare cause of bacterial keratitis. We report a case of a 55-year-old woman presented with pain, redness and profound decrease in vision in both eyes for 2 weeks. One month back she had been treated as acute follicular conjunctivitis elsewhere. She had been treated with ofloxacin drops. On examination, she had central oval fullthickness infiltrate with thinning of cornea and hypopyon in both eyes. She had pseudomembrane in the tarsal conjunctiva. Corneal culture, done separately, showed isolation of Moraxella species, which was resistant to fluoroquinolones. She responded to fortified amikacin and ulcer healed with best-corrected vision of 6/24 and 6/18 in right and left eye respectively. Moraxella keratitis can cause severe keratitis. Conjunctivitis may be complicated by keratitis. Antibiotic resistance can cause problem. Bilateral keratitis should be referred promptly to higher centers if not responding well to treatment.

## INTRODUCTION

Bacterial keratitis, an ocular emergency, is a common cause of corneal blindness. Prognosis depends on the virulence and drug sensitivity of organisms. Common causative agents are streptococcus, staphylococcus and pseudomonas species.^1^ Moraxella species are rare cause of keratitis accounting for 0.3 to 5% of bacterial isolates in different countries.^2-3^ Moraxella are gram-negative, aerobic, diplobacilli, found as commensals in oropharynx, mucous membranes and skin and can cause systemic infections. They can cause keratitis, angular blepharitis, endophthalmitis^2-3^ and in children,conjunctivitis.^4^

We present a case of simultaneous bilateral Moraxella keratitis (MK) in a middle-aged healthy woman with history of conjunctivitis preceding the keratitis.

## CASE REPORT

**Figure 1.**
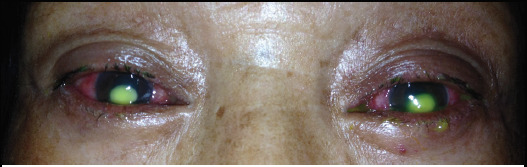
Round central dense corneal infiltrate in both eyes.

A 55-year-old housewife from hilly region of Nepal presented with complaints of redness, pain and watering for one month. In the last 2 weeks, she was having increased pain and profound diminution of vision and white lesion. These complaints were in both eyes. Initially she had been treated as viral conjunctivitis with topical flurbiprofen and ofloxacin, both four times a day. Her documents showed conjunctival congestion, follicles in the inferior palpebral conjunctiva, but clear cornea initially. There was no family history of red eye of the similar kind. She never had purulent discharge. For the last two weeks, her symptoms worsened rapidly with profound loss of vision and she came to our institute. She did not have history of trauma or any other ocular problem. Nor she had any systemic illness. She was non-alcoholic and non-smoker. On examination, vision was hand movements in both eyes. Her eyelids were mildly swollen; there was diffuse conjunctival and circumciliary congestion. Pseudomembrane was present in the upper tarsal conjunctiva. In both eyes there was yellowish oval corneal infiltrate in the centre of cornea; the infiltrate was full thickness with stromal tissue loss and epithelial defect. Margins were distinct (Figure 1).

**Figure 2A, 2B.**
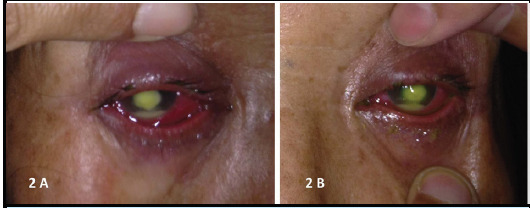
Presence of hypopyon in right eye (2A) and left eye (2B).

Infiltrate size measured about 4mm in both eyes. There was 2mm hypopyon in each eye (Figure 2 A & B).

Corneal sensation was intact. Lacrimal drainage system was patent and B scan was normal. Clinically it looked like bacterial keratitis. Pseudomembrane was removed with sterile forceps. Diagnostic corneal scraping was done for smears and culture sensitivity separately for each eye.

**Figure 3.**
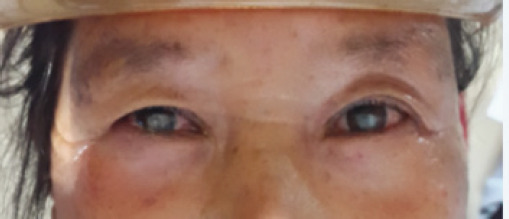
Corneal scar (nebulo-macular) in both eyes at 8 weeks.

Both eye showed Gram negative bacilli in smears. Culture from both eyes grew Moraxella, sensitive to cefazolin, cephalexin, amikacin, gentamycin, tobramycin, chloramphenicol, cefixime, ceftazidime, tetracycline and resistant to ciprofloxacin, ofloxacin, moxifloxacin, gatifloxacin. This could be the reason why ofloxacin given initially did not benefit her from contracting the secondary infection in cornea. Patient was given fortified amikacin one hourly and homatropine three times a day. In the first week, the ulcer did not show improvement. Thinning of corneal was progressive. Oral acetazolamide 250 mg twice a day was added to keep the eye on hypotony to prevent perforation. On the second week, there was improvement with decrease of hypopyon , infiltrate and defect. Antibiotics were tapered to 2 hourly then 3 hourly. By fourth week hypopyon and epithelial defect was gone. Antibiotics were stopped by six weeks. Patient was left with nebulo-macular scar in both eyes (Figure 3).

Vision in right eye was 6/36 unaided, corrected to 6/24 with -1 Diopter sphere (DS). In the left eye, vision was 6/24 unaided, corrected to 6/18 with -0.75 DS. Near vision was N12 with +3.00DS. Optic disc and macula was normal looking. Intra ocular pressure by air puff measured normal readings. Patient was counseled for optical keratoplasty for better vision, but she did not show interest.

## DISCUSSION

Moraxella are rare cause of keratitis. In Nepal, it accounts for 0.54% of bacterial isolates in keratitis.^1^ Most of the case series of Moraxella had shown affection of older age group with average age of 58 to 70 years with slight female preponderance.^2-3,5-6^ In our study, the patient had been middle age healthy female. In older case studies, the predisposing factors for MK had been chronic alcoholism, malnutrition and diabetes but most of the recent studies showed other risk factors like blepharitis, trauma, contact lens and ocular surface diseases.^2-3,5,7^ Predisposing factor had been present in 78 to 87.2% of cases of MK.^3,5,8^

In our patient, ocular surface problem had been the predisposing factor. She had pseudomembranous conjunctivitis with follicles prior to having bilateral keratitis. Viral conjunctivitis was thought of as the etiology on the basis of clinical examination, history and previous documents. Corneal affection in viral conjunctivitis is well known. According to a recent study, pseudomembranous conjunctivitis was associated with subepithelial infiltrates (16.7%), corneal erosion (20.8%) and filamentary keratitis (3.5%).^9^ Corneal affection of viral conjunctivitis could have been complicated by MK in our case. Question can arise whether initial conjunctivitis was bacterial (Moraxella) rather than viral. The most common bacterial isolates in acute purulent conjunctivitis were Staphylococcus aureus, Streptococcus pneumonia, and H influenzae; Moraxella conjunctivitis in adults is rare.^4^ One report mentioned that Moraxella can be a cause for chronic follicular conjunctivitis.^10^ In our patient, since conjunctival swab culture and or polymerase chain reaction test were not done, the exact etiology of conjunctivitis is inconclusive.

Various case series showed that, in more than 80% of MK, the location of infiltrate was either central or paracentral.^7-8^ In most of the studies, the shape of ulcer was oval or round.^3,6-8^ Ring like infiltrate and ameboid pattern is also reported.^2^ Hypopyon is common occurring in 47.7% to 62.5% of MK^2-3,7^ In our study, species identification is not performed. In the case series of McSwiney TJ^6^ and Inoue H,^2^ the most common Moraxella species isolated from keratitis were M. nonliquifaciens and M. lacunata. MK was found to be associated with other types of bacteria in 7.3% to 20 % of keratitis.^2,5-6,8^

It was found that Moraxella isolates have very good sensitivity with fluoroquinolones in most case series^2-3,5,6^ but in our case, it was resistant to them. Das S^5^ and Inoue H^2^ mentioned good responses with amikacin. McSwiney TJ^6^ mentioned all cases sensitive to cefuroxime. Garg P^8^ mentioned 45 % resistance to cefazolin. All cases healed well in the study of Inoue H^2^ but other studies reported need of keratoplasty in 1.2% to 15.4% and evisceration in 3.1 to 7.3%.^3,6-8^

MK, as any bacterial keratitis should be timely and properly treated to avoid complications. Careful follow up of any conjunctivitis is important if there has been corneal involvement with epithelial defect. Prophylactic antibiotics should be in such a situation to prevent secondary infection. Fluoroquinolones resistance as seen in our case may pose a problem and, hence, monitoring of antimicrobial susceptibility would be beneficial.
